# T‐tubule remodeling and increased heterogeneity of calcium release during the progression to heart failure in intact rat ventricle

**DOI:** 10.14814/phy2.13540

**Published:** 2017-12-26

**Authors:** Jasleen K. Singh, Varderes Barsegyan, Nikhil Bassi, William Marszalec, Shannon Tai, Shruthi Mothkur, Maaz Mulla, Elsa Nico, Yohannes Shiferaw, Gary L. Aistrup, John Andrew Wasserstrom

**Affiliations:** ^1^ Department of Medicine (Cardiology) The Feinberg Cardiovascular Research Institute Northwestern University Feinberg School of Medicine Chicago Illinois; ^2^ Department of Physics and Astronomy California State University Northridge California

**Keywords:** Ca^2+^ release, Ca^2+^ transients, heart failure, hypertension, transverse tubules

## Abstract

A highly organized transverse‐tubule (TT) system is essential to normal Ca^2+^ cycling and cardiac function. We explored the relationship between the progressive disruption of TTs and resulting Ca^2+^ cycling during the development of heart failure (HF). Confocal imaging was used to measure Ca^2+^ transients and 2‐D z‐stack images in left ventricular epicardial myocytes of intact hearts from spontaneously hypertensive rats (SHR) and Wistar‐Kyoto control rats. TT organization was measured as the organizational index (OI) derived from a fast Fourier transform of TT organization. We found little decrease in the synchrony of Ca^2+^ release with TT loss until TT remodeling was severe, suggesting a TT “reserve” characterized by a wide range of TT remodeling with little effect on synchrony of release but beyond which variability in release shows an accelerating sensitivity to TT loss. To explain this observation, we applied a computational model of spatially distributed Ca^2+^ signaling units to investigate the relationship between OI and excitation‐contraction coupling. Our model showed that release heterogeneity exhibits a nonlinear relationship on both the spatial distribution of release units and the separation between L‐type Ca^2+^ channels and ryanodine receptors. Our results demonstrate a unique relationship between the synchrony of Ca^2+^ release and TT organization in myocytes of intact rat ventricle that may contribute to both the compensated and decompensated phases of heart failure.

## Introduction

The transverse tubule (TT) network of a ventricular myocyte is essential for conducting the action potential from the surface of the cell into the cell center (Brette and Orchard 2007). These sarcolemmal invaginations play a key role in excitation‐contraction (EC) coupling, ensuring that synchronized contraction occurs following synchronous Ca^2+^ release (Guo et al. 2013; Wei et al. 2010). TTs are spaced at regular intervals of about 2 microns along the surface of the cell and allow for entire cells, and thus the heart, to contract in a coordinated manner (Song et al. 2005). Ca^2+^ signaling occurs in the dyadic junction space where L‐type Ca^2+^ channels (LCCs) are in close proximity to clusters of ryanodine receptors (RyRs). Signaling occurs when Ca^2+^ entry due to an LCC opening initiates a local rise in Ca^2+^ concentration which induces a coordinated release of the local RyR cluster. This signaling is mediated via Ca^2+^‐induced‐Ca^2+^ release and leads to local release events referred to as Ca^2+^ sparks (Oyehaug et al. 2013; Zhang et al. 2014). The TT network facilitates EC‐coupling by allowing LCCs to reach RyR clusters deep inside the cell. Thus, during EC‐coupling Ca^2+^ release occurs throughout the 3‐D volume of the ventricular myocyte. This signaling process is disrupted when the TT network becomes altered so that the spatial relationship between LCC and RyR channels is modified (Brette and Orchard 2007; Guo et al. 2013). Such TT remodeling is seen in the progression from hypertension to heart failure (HF), and is characterized by a decrease in the quantity and density of TTs present, an increase in longitudinal tubules from relatively low levels in control to progressively higher levels coinciding with disease development, and an overall more highly disordered TT network (Guo et al. 2013). As a result of TT remodeling, EC coupling becomes increasingly dyssynchronous, resulting from orphaned RyRs that have become physically separated from LCCs and are consequently activated only by diffusion of Ca^2+^ arising from a longer distance (Guo et al. 2013; Louch et al. 2010). This effect leads to Ca^2+^ release that is delayed in regions containing decreased TT density, which results in EC decoupling (Guo et al. 2013; Louch et al. 2010; Song et al. 2006).

Researchers have been increasingly investigating the subcellular structural basis for the development of HF resulting from a hypertensive state. As previously reported, changes in Ca^2+^ transient characteristics, such as slowed time to peak, as well as diminished TT density have been seen prior to the onset of symptoms of HF (Shah et al. 2014). In the present study, we aimed to further examine the nature of the relationship between TT remodeling and variability in Ca^2+^ release characteristics. Given prior reports of the progressive nature of the transition from hypertension to HF and the role of TT remodeling in Ca^2+^ cycling (Guo et al. 2013; Wei et al. 2010; Zhang et al. 2014; Louch et al. 2010; Shah et al. 2014; Kapur et al. 2010; Aistrup et al. 2013; Lyon et al. 2009; Louch et al. 2004; Heinzel et al. 2008; Louch et al. 2006), we wanted to use linescan imaging techniques to allow for a detailed analysis of the ways in which TT remodeling affects the synchrony of Ca^2+^ release which is critical to both contraction and relaxation phases of the cardiac cycle. To complement our experimental observations we have also applied a computational model of Ca^2+^ cycling within spatially distributed dyadic junctions (Restrepo et al. 2008). Using this model we found that Ca^2+^ release heterogeneities are due to the nonlinear dependence of Ca^2+^ signaling on the spacing between LCCs and the RyR cluster within a dyadic junction. These results will help to define the essential relationship between TT remodeling and the disruption of EC‐coupling that is so critical to the progression to HF.

## Methods

The purpose of this study was to examine the relationship between TT remodeling and changes in Ca^2+^ cycling along the cell length, specifically focusing on sarcoplasmic reticulum (SR) Ca^2+^ release. In order to investigate this relationship in situ, we compared TT organization and Ca^2+^ release characteristics in individual ventricular myocytes in intact hearts of spontaneously hypertensive rats (SHRs) and Wistar‐Kyoto rats (WKYs) between 4 and 22 months of age. All animals were treated in accordance with the protocol approved by the Institutional Animal Care and Use Committee at Northwestern University, as dictated by National Institute of Health guidelines.

### Measurements of TT organization by fluorescence confocal imaging

Imaging of individual ventricular myocytes for the measurement of TT organization was achieved through a procedure that we have described previously (Wei et al. 2010; Shah et al. 2014; Aistrup et al. 2013; Wasserstrom et al. 2009). A Langendorff apparatus located on the stage of a Zeiss LSM510 laser scanning confocal microscope was utilized for this procedure. The heart was removed from an anesthetized rat and perfused with modified Tyrode's solution (0.25 mmol/L CaCl_2_) at a stable pressure of 60–100 mmHg. Re‐circulation was initiated and the heart was stained with di‐4‐ANEPPs (3–5 *μ*mol/L) and Fluo‐4AM and contraction was abolished with a combination of cytochalasin‐D (60 *μ*mol/L) and belbbistatin (25 *μ*mol/L). *z*‐stack images showing the organization of TTs within subepicardial left ventricular myocytes were acquired and two‐dimensional (2‐D) images were analyzed to obtain a value representative of the level of organization within individual cells. An organizational index (OI) value was determined using ImagJ software to select the entire intracellular area, excluding the cell membrane, then further analyzed using customized Matlab software to produce an output value representative of the level of organization within the cell, also taking disorganization – including axial tubules – and any background signal into account. Each cell image was converted to a fast Fourier transform which served as the basis for calculation of OI as the fraction of the organized TT signal compared to the total of organized and disorganized TTs. OI values were measured in a range of approximately 0.4–0.95, representing low to high levels of TT organization, respectively (Shah et al. 2014).

### Simultaneous calcium transient/OI measurements

For this study, OI values for individual myocytes were compared to Ca^2+^ transient measurements made in those same myocytes. As mentioned above, Ca^2+^ indicator was loaded via recirculating perfusion of the heart with Tyrode's solution then adding three increments of Fluo‐4 AM, after which fresh Tyrode's solution (CaCl_2_ = 1.8 mmol/L) was used to wash the heart for 20 min. Recirculation was restored and included cytochalasin‐D and blebbistatin. Basal pacing at a basic cycle length (BCL) of 700 msec was established via the placement of silver needle electrodes in the apex of the left ventricle. To allow for comparison of TT organization to intracellular Ca^2+^ transients, longitudinal linescan images for each myocyte were recorded for analysis after a 2‐D image for TT measurements was recorded during basal pacing and during rapid pacing. Measurements of F/F0 were obtained using a locally developed Matlab program for Ca^2+^ transient measurements in which F/F0 was measured for each pixel. In these experiments, the main parameter of interest was the time of rise to 50% of the peak of the transient (TR_50_), as a measure of rate of SR Ca^2+^ release (Shah et al. 2014). It is important to note that questions have been raised about membrane and intracellular dye distribution in the use of both membrane adsorbing and Ca^2+^‐sensitive dyes in order to show real heterogeneities in membrane distribution or Ca^2+^ release in different cellular regions. We have used membrane dyes for many years and have always observed a smooth transition in TT density and calculated organizational index (OI) with no sudden loss of TT density indicating a threshold effect with disease progression that would indicate that some minimal TT opening size would somehow limit dye access to the TTs. As far as uniformity of Ca^2+^‐sensitive dye distribution is concerned, it is always quite uniform in normal cells and there is no reason to think that it distributes differently in diseased myocytes. This idea is supported by our observations (see Results) that Ca^2+^ transients are quite uniform at low rates even in myocytes from diseased hearts and it requires rapid pacing to really highlight the differences in release that are unrelated to dye distribution and reflect real differences in release along the cell length.

### Measurement of TR_50_

Linescan images were analyzed using a Matlab program that measured multiple parameters for Ca^2+^ transients, including time from initiation to the start of the time to 50% width for each sarcomere at 2 micron intervals (approximating the length of a sarcomere) along the entire cell length, which was used to calculate the TR_50_ at each interval. The linescan images chosen for each cell were obtained at the fastest pacing in which transients had a reasonable signal‐to‐noise ratio (usually 300 msec cycle length). The overall range of cycle lengths used in this study was 150–400 msec depending on the severity of disease. For the analysis, two representative transients were averaged for each cell, with cycle lengths chosen to exclude high levels of background noise, spontaneous waves, and Ca^2+^ alternans. After adjusting the baseline, individual sarcomeres were culled if they had an unacceptably low signal‐to‐noise ratio, were highly divergent from the mean, or had double peaks. Subsequently, the program converts *F* versus *T* to *F*/*F*_0_ versus *T*. In order to avoid over‐smoothing of Ca^2+^ transients, the program incorporated a default low‐pass filter (50 Hz). The analysis was then completed and an output of various parameters was exported to an excel file upon verification that the program correctly identified the initiation and peak of the mean transient and the transient of an individual sarcomere. TR_50_ values were calculated for each of the two sequential transients chosen for an individual cell by calculating the difference between the initiation time of the transient and the time to 50% width for each sarcomere. The standard deviation of these values was recorded as the TR_50_ HI (heterogeneity index) (Wasserstrom et al. 2008).

### Statistical analysis

Data were fitted to either a linear or exponential fit for the graphs of TR50 variability at different basic cycle lengths. The goodness of fit was assessed as *r* ^2^ values and *P* values for the fit was obtained using Sigmaplot V11.

### A spatially distributed model of subcellular Ca^2+^ cycling

**Figure 1:**
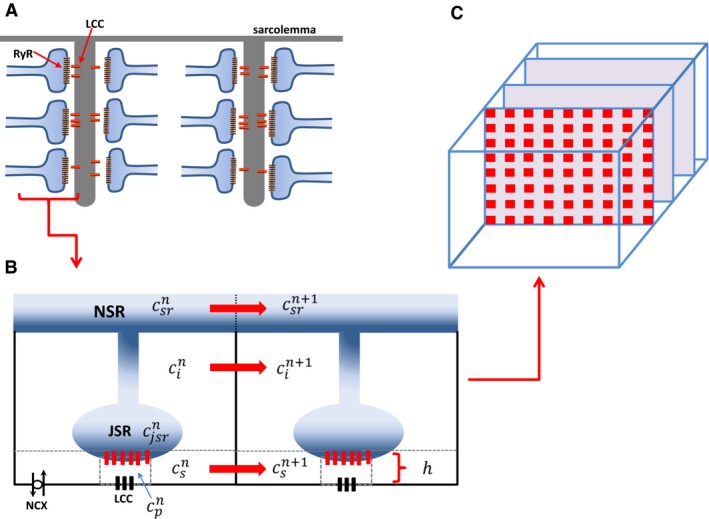
(A) Schematic illustration of the spatial architecture of Ca^2+^ signaling in a cardiac ventricular cell. Signaling between channels occurs within dyadic junctions distributed in the 3‐D volume of the cell. (B) Illustration of two nearest neighbor signaling units (Ca^2+^ release units [CRUs]) showing the subcellular compartments. Here, the superscript n denotes the $n^{\mathrm{th}}$ CRU in a 3‐D grid representing the cell. The spacing between LCC and RyR channels is denoted by the variable h. (C) Spatial architecture of the cell interior showing *Z*‐planes.

To model the spatiotemporal distribution of Ca^2+^ in ventricular myocytes we have implemented an established mathematical model developed by (Restrepo et al. 2008; Shah et al. 2013). In this model, the myocyte is represented as a collection of subcellular compartments that are distributed in a 3‐dimensional (3‐D) representation of the cell interior (Fig. 1A–C). To model the spatial distribution we denote the Ca^2+^ concentration in compartment *x* as $c_{x}^{n}$ (Fig. 1B), where the superscript *n* indicates the location of that compartment in a 3‐D grid representation of the cell interior. In this study we will label our units according to the scheme *n* = (*n*_*x*_, *n*_*y*_, *n*_*z*_) where *n*_*x*_ denotes the longtitudinal direction, *n*_*y*_ is the width of the cell, and *n*_*z*_ is the height. The subcellular compartments in the model are: (1) The dyadic junction, with concentration $c_{p}^{n}$, where a few LCCs on the cell membrane are in close proximity to a cluster of ∼100 RyR channels attached to the junctional SR (JSR). In this model the distance between the LCC channels and the RyR cluster is denoted by the variable *h*, which will be varied to model various states of HF (Fig. 1B). (2) The submembrane space, with concentration $c_{s}^{n}$, which represents a volume in the vicinity of the sarcolemma, regulates membrane‐bound ion currents such as the NaCa exchanger and LCC. (3) The bulk myoplasm, with concentration $c_{i}^{n}$, which characterizes the volume of space into which Ca^2+^ diffuses before being pumped back into the SR via SERCA (Sarcoplasmic‐endoplasmic Reticulum Calcium ATPase). (4) The junctional SR, with concentration $c_{\mathrm{jsr}}^{n}$, that is the portion of the SR network that is positioned close to the cell membrane; (5) The network SR (NSR), with concentration $c_{\mathrm{nsr}}^{n}$, which represents the bulk SR network that is spatially distributed in the cell. In this study, our cardiac cell model will consist of 60 planes representing *Z*‐planes, where each plane contains an array of 20 × 20 regularly spaced compartments (Fig. 1C). Ca^2+^ diffusion in the cell interior is modeled by allowing a diffusive flux between nearest neighbor compartments of the submembrane, the bulk myoplasm, and the SR network. This diffusive flux between nearest neighbors *i* and *j* has the form $J_{d}^{\mathrm{ij}}=\Delta c_{\mathrm{ij}}/\tau_{\mathrm{ij}}$, where ∆*c*_*ij*_ is the concentration difference between the compartments, and *τ*_*ij*_ is the diffusion time constant. In this study we have kept all parameters the same as in the original (Restrepo et al. 2008) model. Finally, we note that the time evolution of RyR and LCC channels is simulated using established Markov state models (Aistrup et al. 2013), where the stochastic evolution of the channels is computed according to the reaction rates linking the channel states.

### Pacing protocol

In this study, we consider the dynamics of Ca^2+^ cycling when the cell is paced with an action potential (AP) clamp. Our AP clamp is taken to have the simple functional form (Heinzel et al. 2011) given by:

(1) $$V\left(t\right)=\left\{\begin{array}{cc} V_{\min}+\left(V_{\max}-V_{\min}\right)\sqrt{1-{\left((t-m\text{CL})/x\text{CL}\right)}^{2}} & m\text{CL}\leq t\leq m\text{CL}+x\text{CL}\\ V_{\min} & m\text{CL}+x\text{CL}<t<(m+1)\text{CL} \end{array}\right.$$

which mimics an AP between a maximum voltage of *V*_max_ = 0 mV and membrane resting potential of *V*_min_ = −85 mV. Here, the variable CL denotes the pacing period, *m* is an integer denoting the *m* ^th^ paced beat, and *x* = APD/CL. Following previous studies (Heinzel et al. 2011) we let this ratio vary with pacing rate according to the functional form *x* = *a*/(*a* + CL) where *a* = 2/3.

## Results

### Relationship between SR Ca^2+^ release and OI

**Figure 2:**
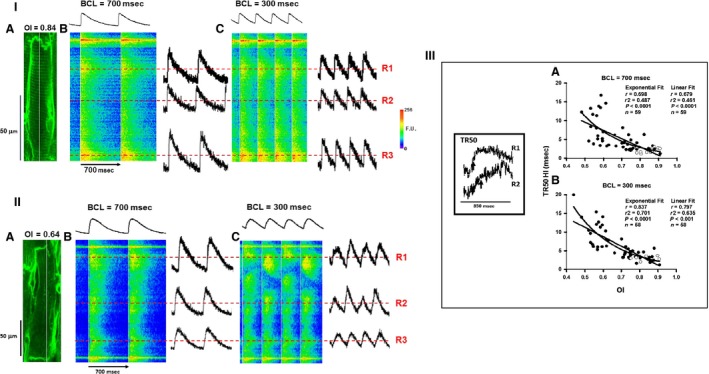
Panel I: Representative 2‐D and linescan images for cells with high value of OI from a 7‐month‐old WKY. (A) 2‐D image of a well‐organized myocyte. The white line indicates the position of the scan line. (B–C) corresponding linescan images showing Ca^2+^ transients (B–C) at BCL = 700 and 300 msec, respectively. Top tracing shows the mean intensity along each line shown in the linescan image below. Three regions (dotted red lines: R1, R2, and R3) are indicated on this image showing representative transients with rapid Ca^2+^ release. The red horizontal dashed lines have been positioned at 50% of peak for both basal and rapid pacing rates where TR50 is measured. Vertical white lines are placed at the initiation of each transient. Panel II **.** TTs and Ca^2+^ transients in a myocyte with severe TT loss from a 12‐month‐old SHR. (A) 2‐D image of a myocyte with poor organization. The white line indicates the position of the scan line. (B–C) corresponding Ca^2+^ transients. Three representative regions (dotted red lines: R1, R2, and R3) are indicated from this image showing representative transients with varying rates of slow Ca^2+^ release. The lines have been positioned at the 50% of peak for both basal and rapid pacing rates. The red horizontal dashed lines have been positioned at 50% of peak for both basal and rapid pacing rates where TR_50_ is measured. Vertical white lines are placed at the initiation of each transient. Panel III: Summary of the relationships between TR_50_ HI and OI during basal (A) and rapid (B) pacing for SHRs and WKY rats (depicted by filled and empty circles, respectively). The inset shows the Ca^2+^ release in two different regions (R1 and R2) of a myocyte from a 12 month SHR similar to that in Figure 3 with the time of TR50) indicated by the vertical arrows. *N* = 4 WKY hearts and 12 SHR hearts.

We have reported previously that TT organization declines in individual cardiac myocytes in intact hypertensive rat hearts and that the number of myocytes showing poor organization increases as myocardial mechanics show signs first of diastolic and then of systolic HF (Aistrup et al. 2013; Shah et al. 2013). Because of the critical nature of the fundamental relationship between cell architecture in the form of TTs and activation of SR Ca^2+^ release during normal EC coupling, there has been a great deal of interest in how TT remodeling affects Ca^2+^ transients, particularly SR Ca^2+^ release. Figure 2 (Panel I) shows a 2‐D image and the corresponding linescans for a myocyte with high TT organization. Figure 2IA shows a 2‐D image of a myocyte from a 7‐month‐old WKY with a high OI value (0.84). The white line in the 2‐D image represents the location in which Ca^2+^ transient measurements were recorded along the length of the cell in subsequent panels. Figure 2IB shows the corresponding linescan image obtained during basal pacing at a relatively low BCL of 700 msec, with the average intensity profile for the transients located immediately above the image. This linescan shows a set of transients in which the individual transients at all sarcomeres are consistent and all rise rapidly followed by a gradual decay during removal of Ca^2+^ from the cytoplasm. The white vertical line indicates the initiation time for each transient and the fact that there is virtually no deviation in fluorescence intensity along the line indicates the uniformity of release along the cell length. Since we would expect that any heterogeneities in SR Ca^2+^ release would be exaggerated during rapid pacing, we also repeated the pacing protocol at a fast rate in this heart (BCL = 300 msec, Figure 2IC). Virtually identical results were obtained during rapid pacing where synchronous and rapid activation of the transient was achieved along the entire cell length. The three cellular regions (indicated as R1, R2, and R3 by the dashed red lines) show Ca^2+^ transients of both small and large magnitude in which rise time is rapid and uniform throughout the cell that is independent of magnitude and location.

Figure 2 (Panel IIA) shows a myocyte from a 12‐month‐old SHR with poor TT organization and a highly disrupted TT network comprised of a few scattered TTs (OI = 0.64). Figure 2IIB shows the corresponding linescan image during basal pacing with a small and variable Ca^2+^ cycling along the length of the cell as indicated by the variability in fluorescence intensity to the right of the white line indicating the time of initiation. Heterogeneity of Ca^2+^ release was severe when BCL was reduced to 300 msec (Fig. 2IIC), with highly variable release rates at the three randomly selected regions with slow or delayed release. There is clearly a high degree of variability in release rate all along the release front as indicated by the heterogeneity in fluorescence intensity immediately following the initiation of release (indicated by the white lines). This variability in release rate is further exaggerated during rapid pacing (Fig. 2IIC). These two figures are presented to show representative examples of 2‐D images and their accompanying linescans in order to demonstrate how the level of organization of the TT network affects SR Ca^2+^ release in Ca^2+^ transients. Similar results have been presented by other investigators in isolated myocytes of failing SHRs to those we found here in intact hearts (Song et al. 2006).

Figure 2 (Panel III) shows summary data for all myocytes in which OI and TR_50_ were measured in order to obtain comparisons between OI and the variability in Ca^2+^ release for both SHRs (filled circles) and WKY control rats (open circles). Since WKY rats are considered normal controls, these served to provide a basis for comparison with myocytes studied in the SHR hearts during the development of hypertensive disease. The inset at the left of Figure 2III shows examples of two cellular regions from another myocyte in the same heart as that shown in Figure 2II at an expanded time scale indicating the time when TR50 is achieved (Fig. 2II, vertical black arrows). In these graphs of TR_50_ HI versus OI, the TR_50_ HI (variability in Ca^2+^ release) was fitted to both linear and exponential relationships during both basal and rapid pacing in order to determine the nature of the reliance of SR Ca^2+^ release on TT organization at both slow and rapid heart rates. Note that the goodness of fit is indicated by both the *r* and *r* ^2^ values shown on the summary graphs. We found that there was an overall increase in variability in SR Ca^2+^ release with decreasing OI during basal pacing (Fig. 2IIIA) that was nearly identical in goodness of fit when described by either a linear or exponential relationship. In contrast, during rapid pacing, the relationship was fitted best by an exponential function, the heterogeneity in SR Ca^2+^ release along the cell length increasing exponentially with a decrease in OI (Fig. 2IIIB). In both cases, the high OI values from WKY rats were clustered among similar values of the SHRs, indicating that there was nothing unique about the disease process itself and that uniform Ca^2+^ release occurs in both control and diseased hearts and is related only to OI. These data demonstrate that Ca^2+^ release becomes increasingly variable as the TT network becomes increasingly disrupted and, furthermore, that this relationship becomes progressively more sensitive to decreasing OI in a nonlinear manner at higher heart rates. Interestingly, the relationship described by the exponential fits demonstrates that little variability in release rate occurs until OI falls below about 0.65, below which there appears to be an acceleration in variability in TR50. These effects suggest not only that there is an apparent threshold effect for increased variability in release rate but that variability in release shows little sensitivity over a wide range of declining OI values.

### A computational model of HF progression

**Figure 3:**
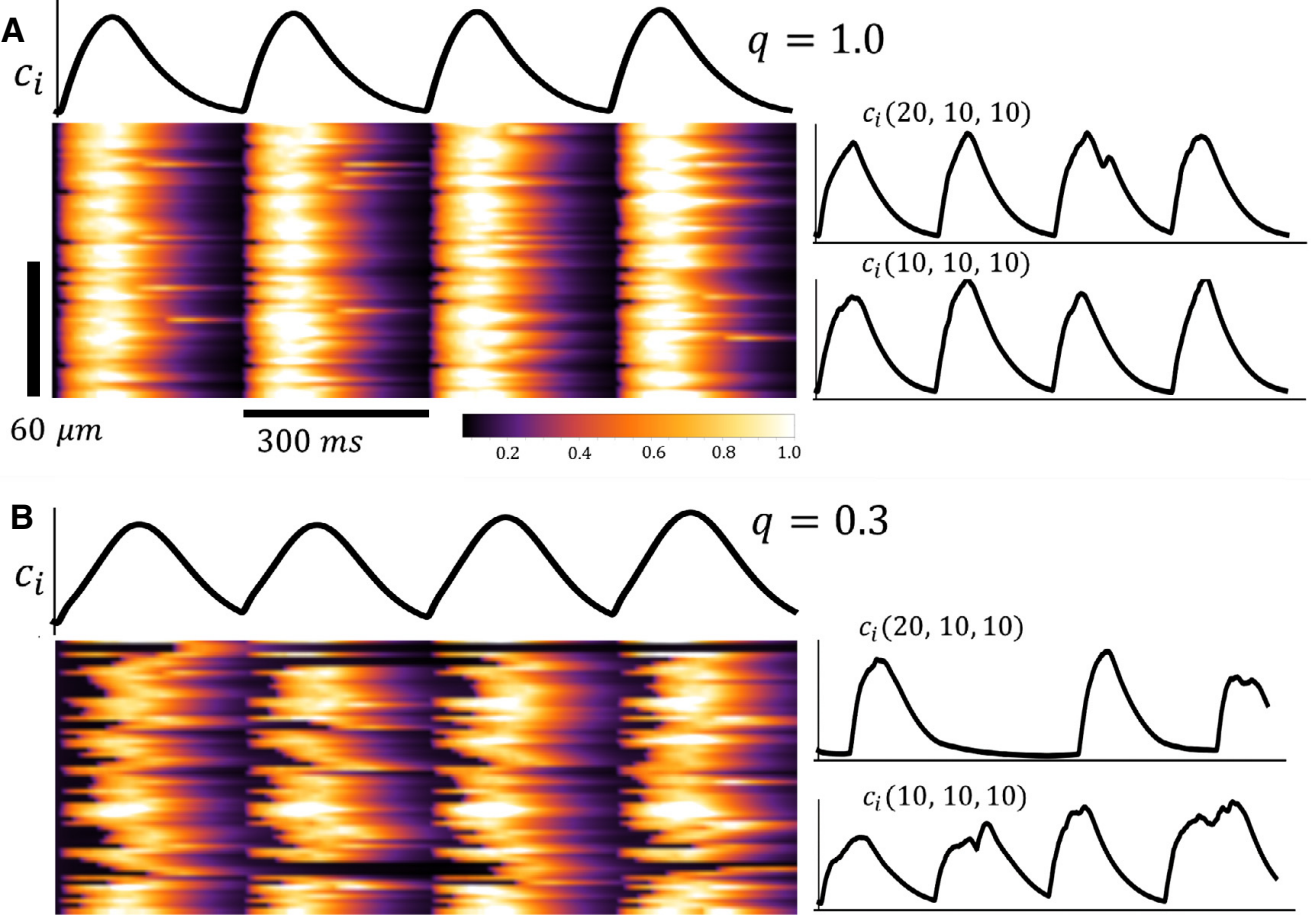
(A) Linescan image of the cytosolic Ca^2+^ concentration $c_{i}^{n}$ when the cell is paced for four beats at BCL = 300 msec. Line scan is taken along the longitudinal direction *n*_*x*_ with Ca release units at $n=\left(n_{x},10,10\right)$. Top trace is the total average cytosolic Ca^2+^ concentration in the cell. Side traces are the cytosolic Ca^2+^ concentration at junctions *n* = (10,10,10) and *n* = (20,10,10). In this simulation the fraction of sites with LCC is *q *=* *1.0. (B) Linescan image and cytosolic Ca^2+^ transients for HF model with *q *=* *0.3.

**Figure 4:**
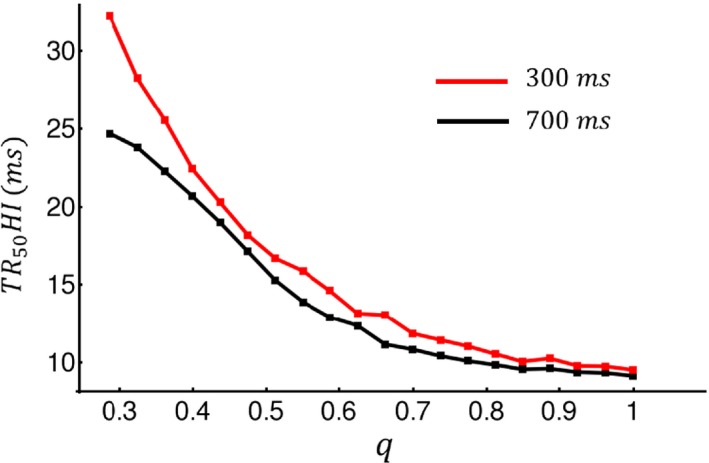
Plot of TR_50_ HI versus *q* at cycle length 300 msec and 700 msec. TR_50_HI was computed by measuring the time to half maximum within 60 junctions along the longitudinal direction. The computed standard deviation was then averaged over 100 independent simulations.

To explore the relationship between t‐tubule organization and Ca^2+^ cycling, we have implemented our spatially distributed computational model of subcellular Ca^2+^ signaling. To model the progression to HF we note that control cells display a highly ordered array of t‐tubules, while in HF cells this system appears highly disorganized. Since t‐tubules are responsible for distributing LCC deep into the cell interior, any disruption in their spatial organization will modify the tight relationship between LCC and RyR clusters. As a starting point we first modeled HF using a simplified approach where we only varied the number of dyadic junctions in which Ca^2+^ signaling occurs between LCC and RyR channels. This is a realistic model since we expected HF to disrupt the spatial organization of dyadic junctions so as to eliminate Ca^2+^ signaling within a population of junctions in the cell. To implement this model we placed LCC only in a fraction *q* of dyadic junctions in the cell. Thus, we varied *q* in the range 0.3 ≤ *q *≤* *1, where *q *=* *1.0 denotes normal Ca^2+^ signaling and where *q* = 0.3 models fully developed HF. In Figure 3A we show a simulated linescan image of the cytosolic Ca^2+^ concentration $c_{i}^{n}$ in response to 4 beats of the AP clamp given by Equation 1. To image Ca^2+^ we plotted $c_{i}^{n}$ along the line *n* = (*n*_*x*_,10,10) where *n*_*x*_ denotes the coordinate along the longitudinal direction. In the same panel (right) we indicate the local Ca^2+^ concentration in the cytosol at two distinct sites in the cell, and in the top trace we show the cytosolic Ca^2+^ concentration averaged over the whole cell. In Figure 3B we repeated the same simulation for the case where *q *=* *0.3. In this scenario we observed that the timing of Ca^2+^ release is substantially more heterogeneous. The global signal (top) appears more blunted as the rise time of the Ca^2+^ transient is increased due to asynchronous Ca^2+^ release. Consequently, the local cytosolic Ca^2+^ concentration exhibits heterogeneities due to the failure of Ca^2+^ release in a large fraction of dyadic junctions. In Figure 4 we plotted TR_50_HI as a function of *q* in order to quantify the relationship between release heterogeneity and the spatial distribution of Ca^2+^ signaling. To compute TR_50_HI we measured the time it takes for $c_{i}^{n}$ to reach half of its maximum. This quantity is computed along the center of the cell and the standard deviation was computed from that population of dyadic junctions. We then averaged this quantity over 100 independent simulations of the whole cell. These computations were performed for cycle lengths of 300 and 700 msec and show that TR_50_HI increases more rapidly with decreasing *q* at 300 msec. Our results indicate that TR_50_HI increases in a nonlinear fashion as q is decreased, which is consistent with our experimental observations shown in Figure 2.

**Figure 5:**
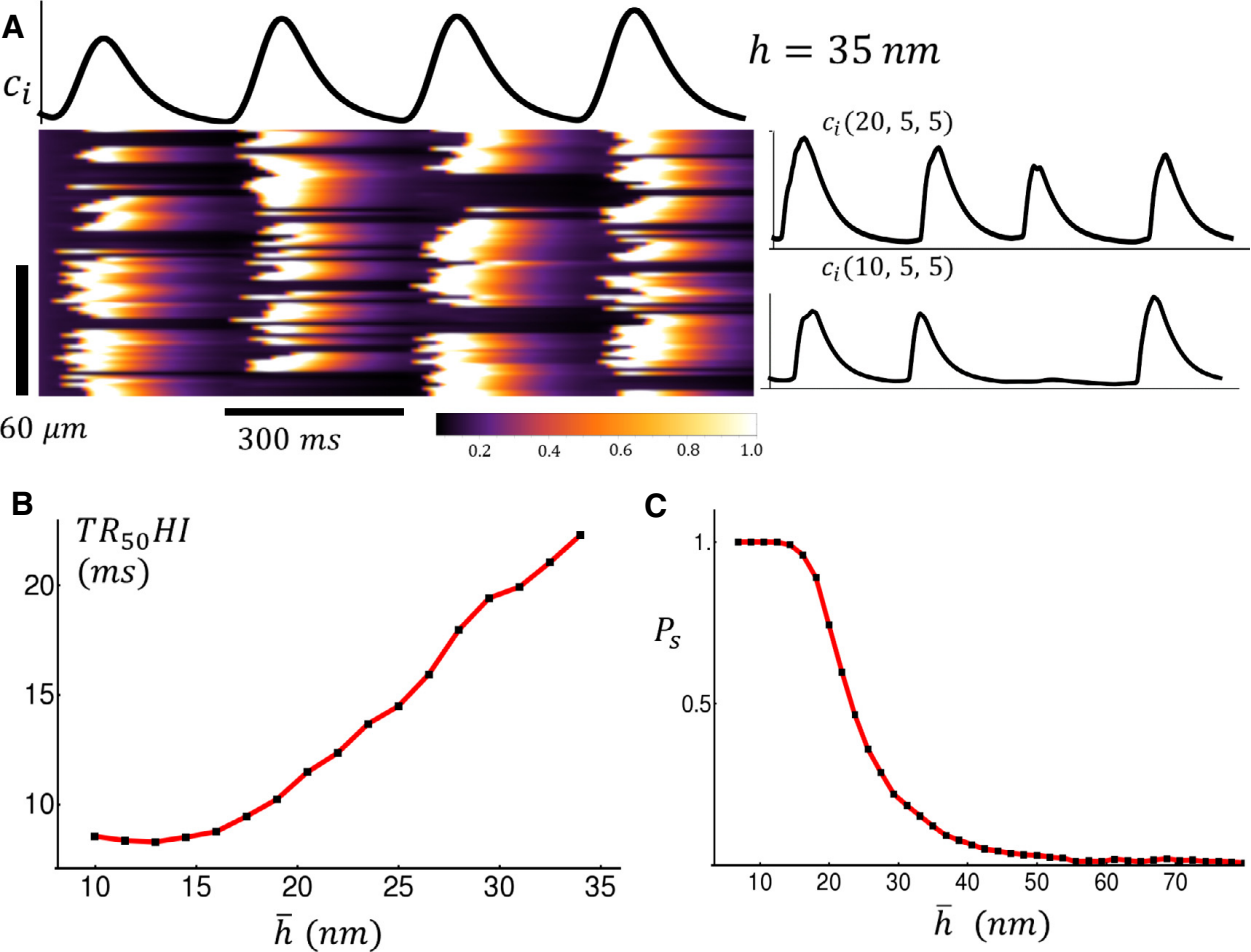
(A) Linescan image of the cytosolic Ca^2+^ concentration. In this simulation the LCC‐RyR average spacing is fixed at $\overset{¯}{h}=30\;\text{nm}$ with standard deviation *σ* = 2 nm. All other parameters are identical to those used in Figure 3. (B) Plot of TR_50_HI versus the average spacing $\overset{¯}{h}$ at BCL = 300 msec. (C) Probability *P*_*s*_ that a Ca^2+^ spark occurs within a dyadic junction in response to a 300 msec action potential (AP) clamp. Criteria for Ca^2+^ spark is that dyadic junction concentration *c*_*p*_ exceeds 50 *μ*mol/L during the 300 msec duration of the AP clamp.

The spatial distribution of LCC and RyR channels under different stages of HF progression is not known in detail. Thus, we applied our model to investigate how release variability depends on the spatial arrangement of LCCs under a broad range of conditions. In this section we considered an alternative model for HF in which the spacing between LCC and RyR channels was varied in order to model the increased separation between Ca^2+^ channels during HF. This is in contrast to our previous model where signaling was completely eliminated at a fraction of junctions. To simulate the spatial variability that is expected in a cardiac cell we picked the spacing h from a Gaussian distribution of the form $P\left(h\right)=\left(1/\sqrt{2\pi \sigma^{2}}\right)\exp\left[-{\left(h-\overset{¯}{h}\right)}^{2}/2\sigma^{2}\right]$, where $\overset{¯}{h}$ is the average spacing between channels, and σ denotes the standard deviation. Using this approach we modeled the progression to HF as an increase in the average spacing $\overset{¯}{h}$. Under normal conditions we fixed $\overset{¯}{h}=10\;\text{nm}$, and model HF progression by increasing $\overset{¯}{h}$ to 30 nm. For simplicity we kept our standard deviation fixed at σ=2nm. In Figure 5A we show linescan images of the cytosolic Ca^2+^ concentration $c_{i}^{n}$ during four beats at BCL=300msec. In this case the spacing was fixed at $\overset{¯}{h}=30\;\text{nm}$. In this simulation we kept q=1.0 so that under normal conditions subcellular Ca^2+^ release is synchronized and similar to that shown in Figure 3A. When the spacing between channels was increased we found that Ca^2+^ release was highly heterogeneous since the coupling fidelity between LCC and RyR is substantially reduced. To summarize this data, in Figure 5B we plot TR_50_HI versus the average spacing $\overset{¯}{h}$ at BCL=300msec. Here, we found that TR_50_HI remains flat for channel spacings in the range 10–20 nm but then increases substantially as the spacing is increased further. Thus, as in the previous model, Ca^2+^ release variability depends on channel spacing in a nonlinear manner.

In order to understand the mechanism for the nonlinear relationship between Ca^2+^ release and t‐tubule organization we analyzed specifically the dependence of Ca^2+^ signaling on the average distance $\overset{¯}{h}$ between LCC and RyR channels. To quantify this relationship we measured the probability that a spark is induced at a dyadic junction as function of the distance $\overset{¯}{h}$. To compute this quantity we drove our cell with an AP clamp and computed the probability that a Ca^2+^ spark occurs in that junction during the 300 msec duration AP. To compute this probability we ran 1000 independent simulations and computed the number of times a spark occurred at a given junction. In Figure 5C we plot the probability $P_{s}$ as a function of the average spacing between channels $\overset{¯}{h}$. Our result shows that $P_{s}$ is a threshold function of $\overset{¯}{h}$ with $P_{s}∼1$ for $\overset{¯}{h}<20\;\text{nm}$ and where $P_{s}$ decreases to $P_{s}∼0$ when $\overset{¯}{h}$ is increased above 20 nm. This result suggests that the signaling fidelity between LCCs and the nearby RyR cluster drops substantially once the spacing between channels is increased above a threshold. This nonlinear relationship indicates that TR_50_HI will be insensitive to $\overset{¯}{h}$ below the threshold (∼20nm) since the number of sparks recruited in the cell remains fixed.

## Discussion

In the transition from a state of hypertension to HF, changes occurring within the cellular TT network are paralleled by changing Ca^2+^ transient characteristics. This decreasing state of TT organization during the development of HF has an impact on EC coupling, and consequently can affect overall heart function. A diminished TT network has been characterized as a contributing factor to phenotypic changes seen during HF, such as Ca^2+^ transients that are slower and have a smaller amplitude; however, these changes have been recently attributed to TT remodeling, rather than detubulation (Brette and Orchard 2007; Oyehaug et al. 2013; Song et al. 2006; Louch et al. 2004; Louch et al. 2006; Litwin et al. 1996; Biesmans et al. 2011; Heinzel et al. 2011; Hohendanner et al. 2013). Unlike previous notions that TT remodeling may be a result of changes that occur during heart failure, this reorganization of TTs is a progressive process that occurs early on, as supported by our data, and contributes to decreased synchrony of Ca^2+^ release (Guo et al. 2013).

### TT remodeling and variability in Ca^2+^ release

TTs are critical to ensuring that action potential propagation into the cell interior is efficient in its activation of the subsequent Ca^2+^ release and contraction of that myocyte. When the TT network is disrupted, EC coupling becomes less coordinated and thus is less efficient and effective (Song et al. 2006; Heinzel et al. 2008; Biesmans et al. 2011; Litwin et al. 2000). As we and others have demonstrated, Ca^2+^ release becomes progressively less uniform as OI decreases. Interestingly, we found that during TT remodeling, there may be no changes in cardiac function although some cells have undergone moderate to severe TT remodeling (Shah et al. 2014). In highly organized myocytes, Ca^2+^ release from the SR occurs nearly instantaneously along the entire cell length, a consequence of the close proximity between LCCs and RyR clusters (Louch et al. 2006). In contrast, Ca^2+^ transients in cells with low levels of organization have a slower rise that is less uniform and more rounded, as different parts of the cell exhibit variable release. The result is that some cell regions begin to contract before other cell regions, causing an overall slowing of time‐to‐peak contraction (responsible for the rounding and delay in contraction) and perhaps as problematic, causing some cell regions to be passively stretched while others are contracting. Our results as well as others (Song et al. 2006) also show that these poorly coordinated Ca^2+^ release events are based upon disorganized TT networks, due to a lack of normal t‐ tubules that have either been replaced by longitudinal elements or empty gaps, leaving orphaned RyRs.

Consequently, given that TT remodeling occurs prior to the onset of HF, changes in Ca^2+^ transient parameters are also seen early on (Wei et al. 2010; Shah et al. 2014; Kapur et al. 2010). This can be attributed to the isolation of LCCs from RyRs; therefore, activation is caused by the diffusion of Ca^2+^ in secondary CICR, a slower process that leads to abnormal EC coupling (Song et al. 2006; Kapur et al. 2010). As supported by this study, as well as by previous findings (Hohendanner et al. 2013), disruption of the TT system results in dyssynchronous Ca^2+^ release. Other studies have also reported that regions lacking a well‐defined TT network also demonstrated delayed and slower Ca^2+^ release, weaker contractions, and Ca^2+^ waves (Louch et al. 2010; Louch et al. 2004; Louch et al. 2006). In addition, a recent study reported that rate‐dependent acceleration of SR release (due to Ca‐calmodulin kinase II activation) is absent in cell regions lacking TTs so it is likely that local elevations in Ca^2+^ are insufficient to induce this form of RyR modulation by LCCs (Dries et al. 2013). This occurs despite maintained SR Ca^2+^ load thus effectively exaggerating the already increased physical distance between RyRs and the nearest LCC. Overall, these effects can be attributed, in part, to fewer Ca^2+^ channels and reduced trigger Ca^2+^ entry resulting from the loss of TTs that contain them (Louch et al. 2010; Lyon et al. 2009).

Based on our findings, the progression in TT remodeling does not appear to significantly affect EC coupling until a critical extent of remodeling occurs at least during rapid pacing, where nonuniformities in SR Ca^2+^ release would likely be exaggerated compared to slow heart rates where Ca^2+^ cycling has more time to recover between beats. This notion of a TT “reserve” is supported by the exponential fit in Figure 2IIIA and B. OI values decline over a very wide range from 0.95 to 0.65 with virtually no change in SR Ca^2+^ release, suggesting that myocytes have undergone extensive TT remodeling but that the degree of TT remodeling is not so severe as to actually interfere with Ca^2+^ release. The myocyte is able to overcome these deficits in triggering, probably by still having enough of a TT network so that relatively few RyRs are outside the sphere of influence of their closest LCC neighbors. As OI values continue to decrease below this range, there is a nonlinear increase in TR_50_ HI values, also explaining why there is an apparent threshold effect arising from the progressive disruption in CICR. Note that our results cannot provide an exact value for a putative threshold for OI below which Ca^2+^ release is affected and above which it is not. Rather, we interpret our results to mean that this nonlinear relationship suggests that there is a range of OI values across which there is little slowing in Ca^2+^ release (thus the concept of a TT reserve) but that when some critical degree of TT remodeling is achieved, the result is a progressively more severe fractionation of Ca^2+^ release along the cell length. Our simulation studies also demonstrate that the accelerating slowing in release occurs because of the 3‐D volume considerations that determine local [Ca^2+^] at the cytoplasmic side of the RyR as the distance to the closest TT and resident LCCs increases in a highly nonlinear fashion as OI decreases.

It is also important to acknowledge that there are other changes that occur during hypertrophic remodeling and HF development that could contribute to abnormal Ca^2+^ cycling. For example, it is well known that mitochondrial Ca^2+^ handling is altered during disease development. In addition, given the importance of nuclear Ca^2+^ fluxes in normal cell function, it is possible that changes in nuclear Ca^2+^ handling might also affect EC coupling, especially during long‐term changes in protein regulation and resulting myocyte function, often referred to excitation‐transcription coupling.

### Ca^2+^ release and TT loss – modeling studies

In this study we applied a detailed computational model of Ca^2+^ cycling in order to uncover the relationship between TT remodeling and EC‐coupling. To model the progression to HF we first varied the fraction of dyadic junctions where Ca^2+^ signaling occurs between LCC and RyR channels. In this case we found that TR_50_HI increased in a nonlinear fashion as the fraction of signaling junctions q was decreased. This result is consistent with our experimental findings which show that TR_50_HI increases with decreasing OI with an effect that is accelerated at smaller OI. Using an alternate approach we have also modeled HF as an increase in spacing between LCC and RyR channels. In particular, we find that the probability that an LCC opening induces a Ca^2+^ spark depends on the spacing $\overset{¯}{h}$ in a nonlinear sigmoid fashion. This threshold relationship occurs because an increase in channel spacing leads to a larger dyadic junction volume, which causes a decrease in the amount of Ca^2+^ sensed by the cytosolic side of the RyRs. Since RyRs transition from the closed to the open state in a Ca^2+^‐dependent manner, the cooperative response of an array of RyR receptors is highly sensitive to the local Ca^2+^ concentration in the dyadic junction. Thus, an increase in dyadic junction volume leads directly to a loss of signaling fidelity between LCCs and RyRs. A consequence of this relationship is that variations of the average channel spacing in the range $\overset{¯}{h}<20\;\text{nm}$ will not influence the whole cell Ca^2+^ release since the function $P_{s}$ is constant in this range. However, when the average junction spacing increased beyond $\overset{¯}{h}=20\;\text{nm}$ then Ca^2+^ signaling in a large population of junctions will fail. This result may explain our observation that Ca^2+^ release heterogeneity is small for large OI but increases in a nonlinear manner when the OI is decreased.

In a cardiac cell we expect that HF progression will lead to multiple changes to the cell architecture and even ion channel distribution and kinetics. Thus, it is likely that HF involves both the loss in signaling within a population of channels, and with a reduction in signaling fidelity due to an increased separation between Ca^2+^ signaling channels. Our numerical findings reveal that both these effects lead to a marked increase in release variability which depends nonlinearly on both the fraction of signaling sites and the spatial distance between sites. Thus, our experimental findings shown in Figure 2 are likely explained by a combination of both factors. Overall, our numerical results indicate that Ca^2+^ release heterogeneity is highly sensitive to the changes in cell architecture that are expected during progression to HF.

## Conclusions

Our results demonstrate that the TT remodeling that takes place during the transition from hypertension to heart failure are responsible for the increased variability in Ca^2+^ cycling within the cardiac myocyte. However, there may also be a threshold up to which TT remodeling may occur without resulting in the development of dyssynchronous Ca^2+^ release and disrupted CICR. Our numerical simulations reveal that this behavior can be due to both a reduced density of active signaling junctions, and also dependence of Ca^2+^ signaling on the spatial separation between ion channels. In particular, we find that the degree of remodeling has to be above a threshold such that signal transduction fails within a large population of dyadic junctions. It is the failure of signaling that causes the increased variability in Ca^2+^ cycling at the more advanced stages of TT remodeling. Finally, our results from these theoretical and experimental observations suggest the idea that there is a TT reserve, which allows TT loss without interfering with Ca^2+^ release. This feature of the TT – Ca^2+^ release relationship may serve as a compensatory mechanism to maintain efficient myocardial performance during the progressive changes in cell ultrastructure and function that occur during disease development. However, as TT remodeling reaches critical levels, the high sensitivity of Ca^2+^ release desynchronization to TT disruption is a likely contributor to diastolic and systolic dysfunction and an overall decline in myocardial performance. Thus, it may ultimately be the TT reserve that is critical for efficient EC coupling at the cellular level during disease progression by maintaining the synchronization of Ca^2+^ release throughout the cell.

## Conflict of Interest

None declared.
